# Liquor Drinking

**Published:** 1871-09

**Authors:** 


### LIQUOR DRINKING.

The chances of life are three times greater in favor of those who are temperate as against those who indulge in spirits; for, taking a hundred thousand of each between fifteen and seventy, thirty-two of the latter will die to every ten of the former. Of the same number of each, seventeen thousand of the intemperate will die before reaching fifty years of age, and only four thousand of those who do not "indulge."

